# Hardness Modulated Thermoplastic Poly(ether ester) Elastomers for the Automobile Weather-Strip Application

**DOI:** 10.3390/polym13040525

**Published:** 2021-02-10

**Authors:** Su Hyeon Jeon, Jae Eon Jeong, Seongkyun Kim, Sungwan Jeon, Jin Woo Choung, Il Kim

**Affiliations:** 1 School of Chemical Engineering, Pusan National University, Busandaehag-ro 63-2, Busan 46241, Korea; tngusdl939@pusan.ac.kr (S.H.J.); jeongjaeeon@pusan.ac.kr (J.E.J.); qock99@pusan.ac.kr (S.K.); 2 Green & Eco-Technology Research Team, Institute of Fundamental & Advanced Technology, Central Research Institute, Hyundai-Kia Motor Company, Uiwang 16082, Korea; sungwan@hyundai.com (S.J.); drchoung@hyundai.com (J.W.C.)

**Keywords:** chain extender, hardness, poly(ether ester), seals, thermoplastic elastomers

## Abstract

As a means of developing new material for automobile weather-stripping and seal parts replacing the conventional ethylene propylene diene monomer rubber/polypropylene vulcanizate, a series of poly(ether ester) elastomers are synthesized. The hardness is modulated by controlling chain extender composition after fixing the hard segment to soft segment ratio. Targeted hardness is achieved by partly substituting conventional chain extender 1,4-butandiol for soybean oil-originated fatty acid amide diol that bears a long chain branch. The crystallinity and phase separation behavior resultant elastomer are also tunable simply by modulating chain extender composition and hard to soft segment ratio.

## 1. Introduction

A growing need in environmental issues has attracted interest in the use of thermoplastic elastomers (TPEs) in a variety of areas, including automotive vehicles, consumer electronics, medicine, foods, cable and wires, and daily commodities [1]. Specifically, in automobiles, TPEs contribute to lighter vehicles for better fuel efficiency, leading directly to reductions in environmental impact through improvement in productivity, energy-saving, and recyclability [2,3,4,5]. The sense of crisis regarding environmental issues is especially strong in the automotive sector, as can be seen in the growth of vehicle categories such as electric vehicles and hybrid electric vehicles. Accordingly, the use of TPEs is kept increasing as a replacement for vulcanized rubber and flexible polyvinyl chloride in many automobile parts except tires, aimed at reducing vehicle weight for improved fuel efficiency and lower CO_2_ and NO_x_ gas emissions, and at achieving greater recyclability, for the sake of environmental sustainability [6].

**Figure 1:**
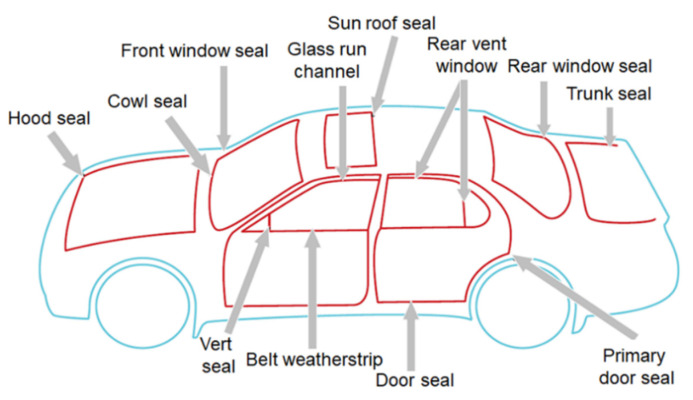
Illustration of seal and weather-stripping parts of automobiles made of ethylene propylene diene methylene rubber (EPDM)/polypropylene thermoplastic vulcanizate (TPV).

Thermoplastic vulcanizate (TPV), dynamically vulcanized ethylene propylene diene methylene rubber (EPDM) and polypropylene, has low compression deforming properties that are similar to vulcanized rubbers. This highly resilient material with excellent tensile strength is characterized by acid, alkali and oil resistance, anti-aging property, weather resistance, and cold/heat resistance (−60 °C to 135 °C) [7]. A broad range of hardness available from Shore 25 A to Shore 70 D adds the value of TPV. Utilizing these features, TPV is widely employed for automotive applications such as door, trunk, and window sealing, including fixed and movable glasses (Figure 1). An average automobile uses 11−16 kg of seals and weather-strip per car. Even though TPV seals both the inside and outside of a car, and has become the best choice to solve existing problems caused by using other elastomeric materials, several regulatory bodies have introduced emission standards that accelerate the usage of lightweight materials to increase fuel efficiency and high-performance. This, in turn, is driving the demand for recyclable thermoplastic poly(ether ester) elastomers (TPEE) on a large scale to manufacture automotive parts where resistance to chemical, heat and oil is required. TPEEs are finding plausible applications in various automobile parts that need to withstand a wide range of temperatures for a very long period.

Considering that this material can be processed with good performance without vulcanizing, we attempted to use it as a new material for automobile seal and weather-stripping parts. Note that this material has intrinsic rubber features of elasticity and durability and can be property modulated for various processings. In this study, we chose TPEEs consisting of poly(tetramethylene ether glycol) (PTMEG) as a soft segment (SS) and poly(tetramethylene 2,6-naphthalene dicarboxylate) as a hard segment (HS). The synthesis of TPEE was first reported by Coleman [8] in 1954. This TPEE block copolymer consists of two separated phases: i.e., crystalline aromatic polyester like poly(butylene terephthalate) (PBT) as an HS and amorphous PTMEG as an SS. PBT-*co*-PTMEG copolymer was first commercialized in 1972 under the tradename of Hytrel by Du Pont (Wilmington, DE, United States) [9]. The morphology [10,11], properties [12], interaction between HS and SS [13], and structure of the crystalline [14,15,16] and amorphous phase [17] have been extensively studied. The effect of filler reinforcement on ageing and compression set properties of elastomers was also systematically studied [18,19].

TPEE is characterized by good tensile strength, good resilience, toughness, wear resistance, oil/chemical resistant, high heat resistance, and high impact resistance [2,3]. The harder the TPEE is, the better its heat resistance is; the lower the temperature, the better its cold resistance is. 

**Scheme 1:**
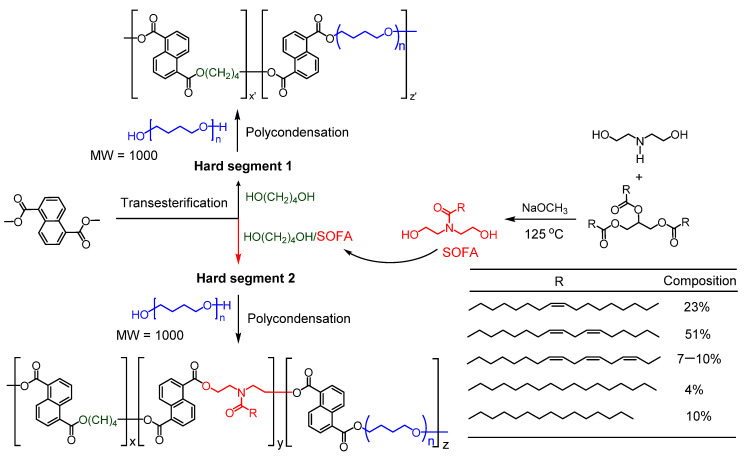
Synthetic strategy of hardness modulated thermoplastic poly(ether ester) elastomers (TPEE) by using chain extender modifier originated from soybean oil.

This material has superior features with its operating temperature ranges from −70 °C to 200 °C. However, the hardness range is in SHORE 30D−SHORE 80D, which is too stiff to apply as a seal and weather-stripping parts. In this study, for the modulation of the hardness of TPEE, we have attempted to employ a new chain extender (CE), soybean oil-originated fatty acid diethanolamide (SOFA), which is prepared directly from the reaction of soybean oil with diethanolamine using the reported method [20], considering the CE becomes HS blocks as illustrated in Scheme 1. SOFA was used as a partial replacement of conventional 1,4-butanediol (BDO) as a CE formulation during the formation of HS by transesterification with dimethyl 2,6-naphthalenedicarboxylate (NDC). The SOFA content varied up to 20% with respect to the BDO content. The resulting HS fragments bearing SOFA on the backbone were further reacted with PTMEG (molecular weight (MW) = 1000). We investigated the effect of SOFA amount on the mechanical behavior, specifically hardness, morphology, and physical properties of the resultant TPEEs.

## 2. Materials and Methods 

### 2.1. Materials

Polymerization grade of NDC, BDO and PTMEG were donated by the Kolon Industries Co. (Gumi, Korea) and used after drying. Irganox 1010, titanium tetrabutoxide (TBT, 97%), sodium methoxide (95%), diethanolamine (98%) were purchased from Sigma-Aldrich (Seoul, Korea) and were used without further purification. Reagent grade of dichloromethane, chloroform, hexane, diethyl ether, and ethanol purchased from Dae Jung Chemical Co. (Daejeon, Korea) were distilled according to standard procedures. A commercial-grade soybean oil purchased from Ottogi Co. (Anyang, Korea) and triflouroacetic acid (TFA) (99%) and 1,1,2,2-tetrachloroethane (97%) purchased from Tokyo Chemical Industries (Tokyo, Japan) were used without further purification.

### 2.2. Synthesis of SOFA

SOFA was synthesized as per the procedure reported in the literature (see also Scheme 1) [20]. Typically, diethanolamine (0.32 mol) and sodium methoxide (0.007 mol) were subjected to agitate using an overhead motor stirrer at 70 °C for 60 min. Soybean oil (0.1 mol) was then added dropwise into the reaction mixture over a period of 90 min with a gradual increase of temperature to 125 °C. The whole reaction mixture was held at 125 °C for next 6 h with constant stirring. The reaction progress was monitored by thin-layer chromatography (TLC). After cooling to room temperature, the reaction mixture was dissolved in diethyl ether and washed with 15% aqueous NaCl solution. The upper organic ethereal layer containing SOFA was separated from the aqueous layer and was dried to obtain SOFA in a 92% yield.

### 2.3. Synthesis of TPEEs Using SOFA as CE

For the synthesis of TPEEs melt polycondensations of NDC and BDO (or BDO/SOFA mixture), and PTMEG were performed in a 600 mL high-pressure stainless steel reactor (Parr Instrument Co., Moline, IL, USA) equipped with a vacuum pump and Dean–Stark apparatus for collecting the by-products [21,22]. TBT (0.3 wt% with respect to NDC) and Irganox 1010 (0.5 wt% with respect to the total mass of monomers) were used as a catalyst and as a thermal stabilizer, respectively. Typically, 33.75 g (0.17 mol) of NDC, 27.53.6 g (0.23 mol) of BDO, TBT (0.15 wt% with respect to NDC), and Irganox 1010 were introduced into the reactor under nitrogen flow. The molar ratio of NDC to CE was maintained at 1:1.5. The transesterification reaction was carried out under a nitrogen atmosphere in the temperature range 160–210 °C for 90 min. By-product methanol was collected and was used to measure the conversion of the transesterification. After the cessation of methanol distillation, 16.67 g (0.016 mol) of PTMEG was added to the reactor along with TBT (0.15 wt% with respect to NDC) as a catalyst. For the polycondensation reaction, the temperature was increased to 250–255 °C, and some of the excess byproduct BDO was distilled off at this stage. Then, the reactor temperature was raised to 260 °C under a high vacuum (0.5 torr), and held for an additional 2h. The resulting TPEEs were dissolved in chloroform at 50–60 °C and purified by precipitating from excess diethyl ether and then was washed with ethanol and vacuum dried at 60 °C for 12 h. Detailed compositional variations are shown in Table 1.

### 2.4. Characterization

The intrinsic viscosity (*η)* of TPEEs were analyzed at 30 °C using a capillary Ubbelohde-type viscometer. The concentration of TPEE was maintained at 0.5 g dL^−1^ in the solvent mixture of 1,1,2,2-tetrachloroethane and phenol (40:60 v/v). The viscosity average molecular weight (*M_v_*) of TPEEs were analyzed by Mark−Houwink equation (*η* = K*M_v_*^a^, where K = 5.36 × 10^−4^ and a = 0.697) [21,22,23]. ^1^H NMR spectra (400 MHz) of TPEEs samples were measured with Varian INOVA 400 NMR spectrometer (Palo Alto, CA, USA). Fourier-transform infrared (FT-IR) spectra of TPEEs samples were measured with Thermo Nicolet iS 5 FT-IR Spectrometer (Thermo Fisher Scientific, Waltham, MA, USA) at room temperature. The spectra of TPEEs samples were recorded at the scanning range 4200–400 cm^−1^. Differential scanning calorimetry (DSC) (Q100 instrument, TA Instruments, New Castle, DE, USA) measurements were carried out in temperature range from −80 to 270 °C and at 10 °C min^−1^ heating rate and −20 °C min^−1^ cooling rate. Dynamic Mechanical Analysis (DMA) was measured on a Q800 dynamic mechanical thermal analyzer (TA Instruments, New Castle, DE, USA) working in tension mode at a frequency of 1 Hz. Then, the TPEEs samples were heated at a rate of 5 °C min^−1^. Glass transition temperatures (*T_g1_* and *T_g2_*) of the TPEE samples were taken as the temperature at the maximum relaxation peak of the loss modulus (*E′′*) and tangent delta (*tan δ*). The rubbery plateau was determined by the storage modulus (E′). Wide-angle X-ray scattering (WAXS) measure of the TPEEs samples using Bruker D8 Advance diffractometer with computerized data collection and analytical tools. The X-ray source CuKα radiation of wavelength *λ* = 1.54 Å was formulated using an applied voltage of 40 kV and a filament current of 40 mA. CuKα radiation was monochromatized with a graphite monochromatizer and Ni filter. The WAXS curves of TPEEs samples were measured in the 2θ range 2–60° with a step size of 0.02°. The hardness was determined with Durometer Shore A type and Shore D type (GS-706N, GS-702N, Teclock, Japan). Stress–strain curves of the TPEE samples were measured with the universal testing machine (KSU05, Kyungsung Testing Machine Co., Ansan, Korea) and the constant cross-head speed was maintained at 100 mm min^−1^. The analysis was performed at room temperature on uniformly shaped specimens. At least five specimens were tested for each given value. The elongation percentage and ultimate strength of TPEEs samples were analyzed with the stress–strain curves. Hardness (Shore A and Shore D) of the TPEE samples were measured based on ASTM D2240 (or ISO 868) using an Automatic Motorized Durometer DigiTest II (Bareiss USA, Inc., Arden, NC, USA). 

### 2.5. Computer Simulations

Mesoscale dynamic simulations of TPEEs structures were all carried out with the MesoDyn package in the Materials Studio 5.0 [24]. MesoDyn utilizes a dynamic variant of mean-field density functional theory (DFT) with Langevin-type equations to investigate polymer diffusion at large length and time scales and a mean-field DFT, using the Gaussian chain as a model, to estimate the thermodynamic forces. Simulations using MesoDyn module were successful used to study several polymer systems [25,26,27]. In MesoDyn simulation, the dimensionless parameters were chosen as follows: the size of cubic grid 32 × 32 × 32 nm, the bond length 1.1543 Å, the bead diffusion coefficient 10^−7^ cm^2^s^−1^, the noise parameter 100.0, the compressibility parameter 10 kT, the simulated temperature 298 K, the time step 0.5 ns. For each system, the total number of steps of 200,000 was carried out to reach a kinetic equilibrium.

## 3. Results

### 3.1. Characterization of TPEEs

**Figure 2:**
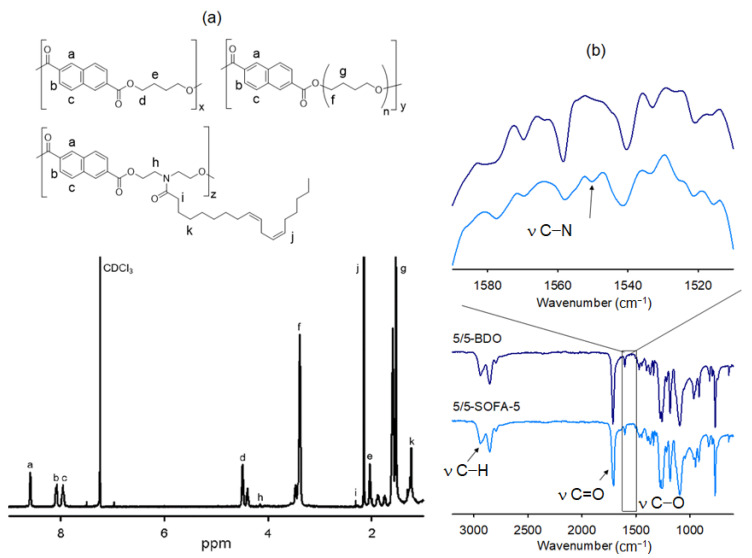
(**a**) ^1^H-NMR spectrum of 5/5-SOFA-5 sample and (**b**) FT-IR spectra of 5/5-SOFA-5 and 5/5-BDO samples.

Considering the modulation of hardness lower than conventional TPEEs in mind, HS/SS ratio was controlled to 50/50 and 40/60 (Table 1). In addition, the molar percentage of SOFA in CE composition was controlled from zero to 20% with respect to BDO. Since the CE fragment becomes HS blocks, the change of CE composition results in the modification of HS blocks (see Scheme 1). The structure of TPEEs bearing SOFA was established by using ^1^H NMR and FT-IR spectroscopies. For example, ^1^H NMR spectrum of 5/5-SOFA-5 (Figure 2a) shows the signals at 7.97, 8.09, and 8.62 ppm assigned to the aromatic protons of the naphthalate moieties of NDC. The chemical shifts at 1.91 and 4.39 ppm are attributed to the methylene protons of the short-chain diol (BDO; CH_2_−CH_2_−CH_2_−CH_2_ and CH_2_−CH_2_−CH_2_−CH_2_, respectively). The signals at 1.63 and 3.43 ppm represent the methylene protons of PTMEG: CH_2_−CH_2_−CH_2_−CH_2_−O and CH_2_−CH_2_−CH_2_−CH_2_−O, respectively [28]. The signals at 2.03 and 4.15 ppm correspond to double bond and methylene proton in SOFA. Accordingly, [BDO]/[SOFA] ratios in TPEE can be estimated using “e” peak/”h” peak area ratio as summarized in Table 1. Controlling *X_SOFA_* with respect to BDO from zero to 20% in feed, *X_SOFA_* in polymer ranges from zero to 11.7%.

FT-IR spectra (Figure 2b) of 5/5-BDO and 5/5-SOFA-5 samples show the characteristic transmittances at 2863, 2947 cm^−1^ (CH_2_), 1718 cm^−1^ [C(O)], 1272 cm^−1^ [C(O)−O], 1118 cm^−1^ (C−O−C), and 769 cm^−1^ (CH_2_). The characteristic peak assigned to C−N in 5/5-SOFA-5 sample appears at 1550 cm^−1^.

As summarized in Table 1, the *η* values of TPEEs range from 1.54 to 2.06 dL/g, which correspond to a range of 101,000 to 156,000 of *M_v_* values. These data clearly show that high MW polymers are successfully formed and show no dramatic changes and conspicuous trends according to compositional changes induced by the variation of polymerization conditions.

### 3.2. Thermal and Viscoelastic Analyses of TPEEs

**Figure 3:**
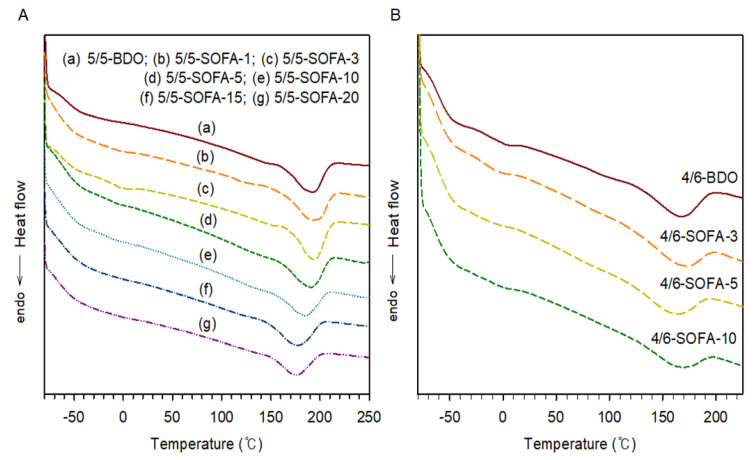
Differential scanning calorimetry (DSC) thermograms of TPEE samples of different HS/SS ratios, 50/50 (**A**) and 40/60 (**B**), prepared by modulating SOFA/BDO ratio. Detailed sample codes are in Table 1.

The versatile properties of TPEE are generally attributed to their microphase-separated morphology, consisting of microdomains rich in HS and a microphase rich in SS, and arising from the thermodynamic immiscibility of HS and SS. The degree of incompatibility is determined to a great extent by the ratio of intra- and intermolecular interactions. TPEE has a SS microphase (*T*_g1_), a blended SS microphase and HS microphase (*T*_g2_), and a HS microphase region (*T*_m_). The *T*_g_, *T*_m_ and *T*_c_ values of TPEEs were determined by DSC (Figure 3) and the results are summarized in Table 1. In a series of TPEE samples consisting of HS/SS = 50/50, the *T*_m_ value of 50/50-BDO sample bearing no SOFA moiety is 195 °C and it decreases monotonously as the relative amount of SOFA increases. The *ΔH_m_* value decreases sharply from 12.38 J/g (5/5-BDO) to 9.19 J/g (5/5-SOFA-20), demonstrating the crystallinity decreases as the number of incorporated SOFA increases. Similar trends are observed for a series of samples with HS/SS = 40/60. 

Considering that TPEE chains are formed by a succession of long flexible blocks (PTMEG) responsible for the elasticity and hard ones formed by transesterification of CE and NDC interconnecting the macromolecules, and each block is incompatible and localized in separate microdomains: i.e., HS and SS microdomains, the replacement of BDO with SOFA bearing bulky alky branch would prevent HS microdomains from close packing. There are three microdomain-separated phases in TPEE samples of this study: (i) an amorphous PTMEG phase where a small, relatively composition-independent amount of independent HS fragments dissolved therein, (ii) an interfacial amorphous HS phase bearing some amount of SS phase, and (iii) a crystalline HS microdomain phase. In the TPEEs with a relatively balanced composition, such as 5/5-BDO and 4/6-BDO, the segment lengths and chemical compositions are sufficient to allow crystallization of both types of segments. As the amount of bulky SOFA integrated into HS blocks increases, the HS amorphous phase increases accordingly due to the difficulty in close packing. Thus, the isolated segments of the other component, especially those species associated with bulky SOFA, to be preferentially dissolved near the boundary or interface formed by the microphase-separated phases. As a result, the segmented TPEEs are multiphase systems with indistinct separation surfaces. In this way, a large amount of SOFA may result in losing elastomeric behavior.

**Figure 4:**
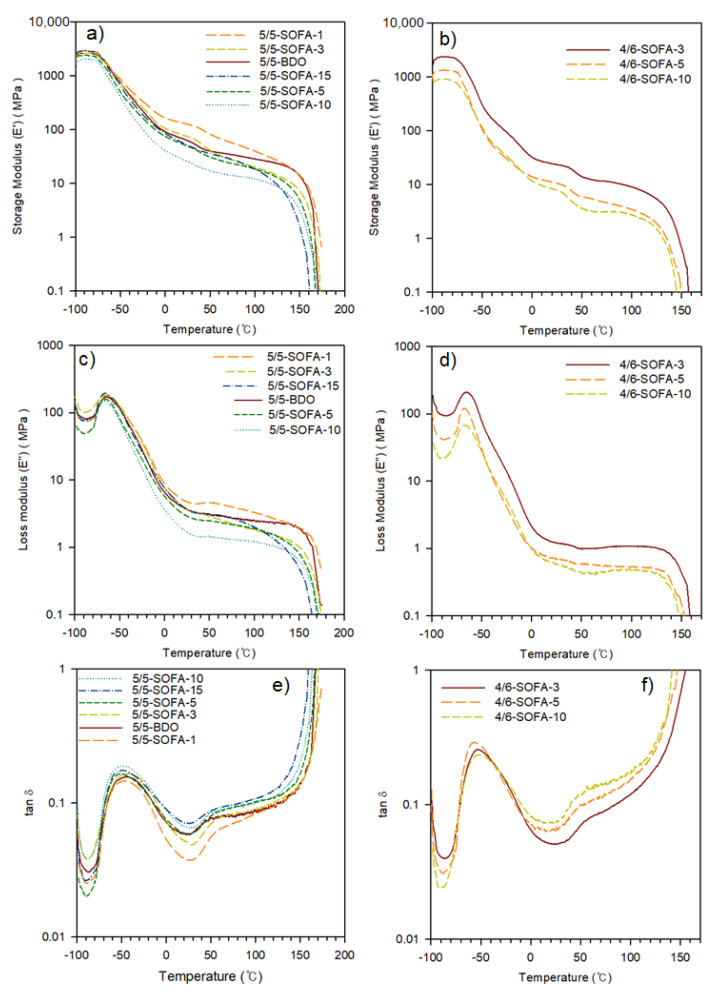
Dynamic mechanical analysis (DMA) scans [storage moduli (**a**,**b**), loss moduli (**c**,**d**), and tan δ (**e**,**f**)] of TPEE samples of different HS/SS ratios prepared by using different SOFA/BDO CE ratios. Sample codes on figures are in Table 1.

The viscoelastic behavior induced by microphase separation was investigated by DMA analysis of the TPEE films of HS/SS=50/50 and 40/60 (Figure 4). The storage moduli of all samples tend to display four particular regions, although there are some additional subtleties. The HS/SS=50/50 samples show initial softening behaviors at around −42 to −49 °C dependent on the amount of SOFA due to the glass-transition behavior of SS blocks. The *T_g1_* (−42 °C) value of 5/5-BDO sample bearing no SOFA moiety monotonously decreases as the number of SOFA increases, resulting in −49 °C for 5/5-SOFA-15 and 5/5-SOFA-20 samples (see Table 1). Interestingly, the reverse trend of the variation of *T_g1_* value was observed for the HS/SS = 40/60 samples. Thus, the *T_g1_* value of 4/6-BDO (−51 °C) slightly increases to −49 °C for 4/6-SOFA-10. A rubbery plateau behavior in the modulus is apparent from about 0 °C to 150 °C, with a reduction to lower temperatures for the TPEEs with higher amounts of SOFA and SS. Considering this general plateau region is typically the service window for many applications, there is a relatively high dependence of stiffness on temperature. 

After the rubbery plateau region, a decline in the modulus is dependent on the melting characteristics of the HS; its onset occurs systematically at lower temperatures as the *T_m_* decreases (lower HS content and lower crystallinity in the same HS). The thermal transition response can be collected more clearly from the corresponding tan δ curves (Figure 4). Transitions induced by the SS blocks and the amorphous moiety of the HS unit can be identified at the lowest temperature region. The glass transition of SS (*T_g1_* as summarized in Table 1) can be confirmed as a second transition [22]. In the third transition, the damping increases again because of the glass-transition behavior of the amorphous fraction of HS blocks. The peak designated as *T_g2_* is particularly distinct and shifts to lower temperatures as SOFA and SS contents increase, due to the fact that the less crystalline HS blocks are possibly further mixed with some of the SS blocks, giving rise to a broadening and a downward shift in their *T_g_*. Finally, tan δ rises simply due to the onset of melting of the crystalline phase HS blocks. As expected, the *T*_m_ value monotonously decreases as SOFA and SS contents increase.

### 3.3. Molecular Dynamics (MD) Simulation

**Figure 5:**
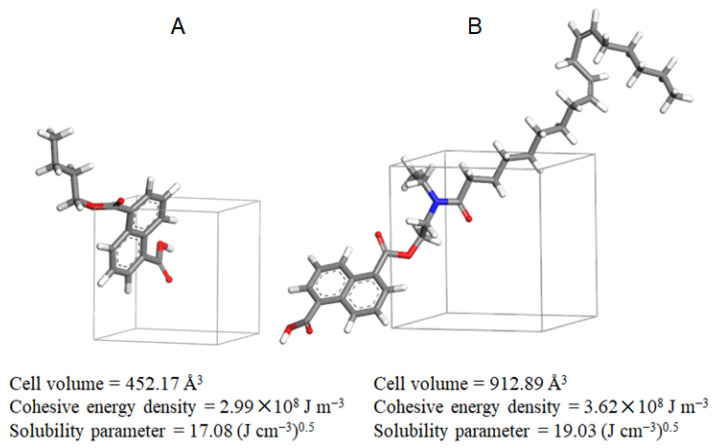
Three-dimensional view of an amorphous cell of HS structural units consisting of 2,6-naphthalenedicarboxylate (NDC) and BDO (**A**) and of NCD and SOFA (**B**). Molecular dynamics (MD) simulations were employed with the Materials Studio. Each HS structural unit was selected to construct the model by the Amorphous Cell module. The time step was set as 1.0 fs and the COMPASS force field was used with the Berendsen algorithm to maintain a constant temperature (298 K) and pressure (100 kPa). After optimizing the molecular structures, 150 ps NPT-MD runs were carried out to further equilibrate the models.

As CE molecules become the units of HS that are implicitly connected to PTMEG SS, the structural difference of CE has a significant effect on the resultant TPEE properties by its ability to drive phase separation and to complement or interfere with regular HS structure, and to promote interhard segment interactions. Comparing amorphous cells of the HS consisting of NDC and BDO with that consisting of NDC and SOFA moiety, the cell volume of the latter (912.89 Å^3^) is larger than that of the former (452.17 Å^3^) by about 2-fold (Figure 5). The structural difference also results in changing polarity and size of their structural fragments, causing the changes in solubility parameter. From the theoretical viewpoint, phase separation can be controlled by the thermodynamic factors which result from differences in structures of HSs and SSs, and by kinetic factors which for example promote separation of PTMEG segments due to their higher mobility and thus lower viscosity of the reaction mixture during polymerization. TPEEs with structural segments derived from NDC/SOFA have lower separation degrees than their equivalent TPEEs obtained from NDC/BDO, due to thermodynamic reasons. The rotation hindering elements, like voluminous substituents at the SOFA moieties, contribute to improved miscibility of phases. Miscibility is also improved by increased polarity (as solubility parameter) of structural fragments within macromolecules. Thus, the micro-phase separation must be less prominent in TPEE bearing HSs from NDC/SOFA due to weaker interactions.

**Figure 6:**
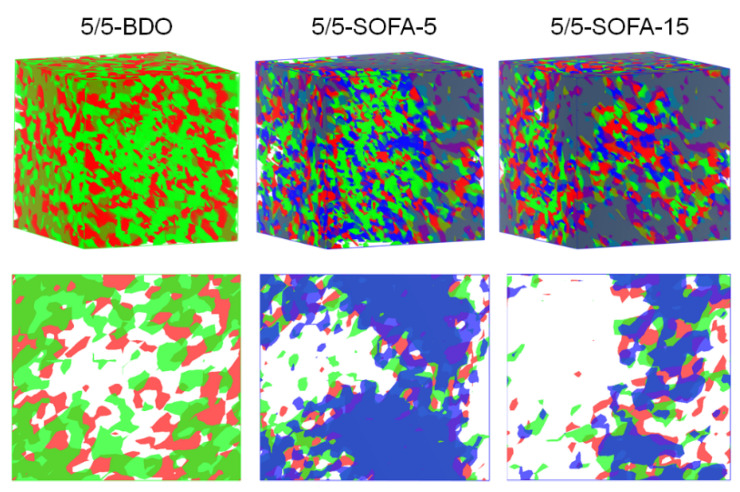
Images of bulk (top) and cross-section (bottom) from MesoDyn simulations of the phase separation of TPEEs synthesized with and without using SOFA. The surface links point at the dividing surface between the two phases. The entire volume is color-coded by the density of one of the blocks (red of HS blocks; green of SS blocks; and grayish to bluish of HS blocks formed by the presence of SOFA of different amounts).

The phase morphology and compositional order parameters, giving a direct measure of phase separation, can be visualized using MesoDyn, a dynamic simulation method for studying the long length and time behavior of complex fluid systems like TPEE by suitably defining interaction energies for each pair of species in TPEEs of different compositions. These energies are proportional to the Flory–Huggins interaction parameter and lead to the phase separation of various components. Firstly we define three chemical species, structural units HS1 comprised of NDC and BDO, HS2 comprised of NDC and SOFA, and SS1 of PTMEG. Considering the molar volume and characteristic ratio of each block and assuming MW of TPEE as 100,000, we performed simulations for three systems: i.e., [(SS1)_7_(HS1)_3_]_50_, [(SS1)_7_(HS1)_3_]_45_[(SS1)_7_(HS2)_1_]_5_, and [(SS1)_7_(HS1)_3_]_40_ [(SS1)_7_(HS2)_1_]_10_. The simulation results based on the effective Flory–Huggins interaction parameters (Table 2) between beads of HS1, HS2 and SS1 clearly show that there are big differences in phase separation according to compositional differences of TPEEs as illustrated in Figure 6. The [(SS1)_7_(HS1)_3_]_50_ system, simulating 5/5-BDO sample, shows a clear and uniform phase separation of each block; however, it becomes bigger and ambiguous as the amount of HS2 beads increases.

### 3.4. WAXD Analysis of TPEEs

**Figure 7:**
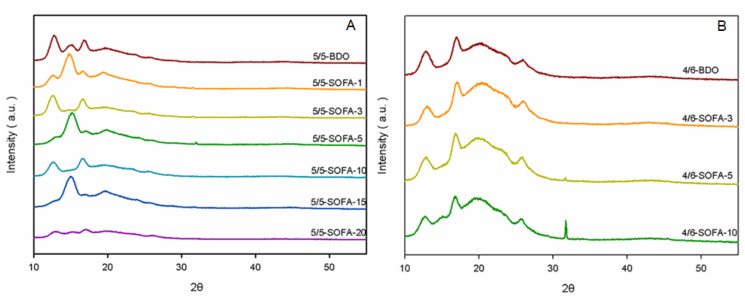
Wide-angle X-ray scattering (WAXS) patterns of TPEE samples of different HS/SS ratios, 50/50 (**A**) and 40/60 (**B**), prepared by using different SOFA/BDO ratios.

Detailed crystalline information was also obtained from the WAXD curves of the TPEE samples annealed at 200 °C for 1 h (Figure 7). The characteristic diffraction peaks of TPEEs bearing no SOFA (5/5-BDO and 4/6-BDO) appear at 2θ = 12.60°, 15.12°, 16.51°, and 19.58° assigned to the (0$\bar{1}$1) (010), ($\bar{1}$01), and (100), respectively with d-spacing of 0.706, 0.587, 0.536, and 0.45, respectively, indicating the α form of the crystal packing structure [30], which is typical of poly(butylene-2,6-naphthalene dicarboxylate) when crystallized from the molten state. The characteristic diffraction peaks become weaken and broaden with increasing SOFA and SS contents, clearly showing that the chemical incorporation of SOFA into the TPEE backbone constrains the close packing of HS due to geometric constraints induced by bulky SOFA units.

### 3.5. Mechanical Properties of TPEEs

**Figure 8:**
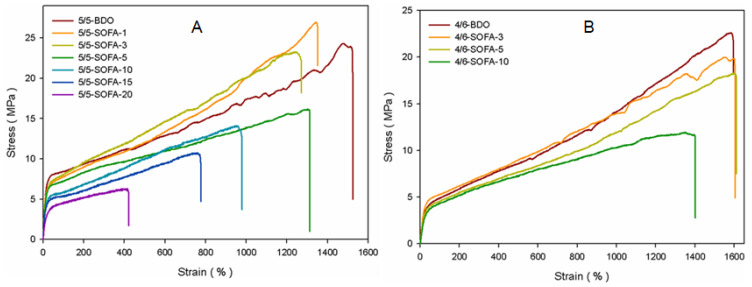
Stress–strain curves of TPEEs with different hard and soft segments (50/50 (**A**) and 40/60 (**B**)) synthesized by using BDO and BDO/SOFA as chain extenders.

The mechanical properties are related to the elongation percentage, ultimate strength and Young’s modulus. Stress−strain curves of TPEEs are shown in Figure 8 and the results are summarized in Table 3. Since the composition of HS usually provides strength, modification of them with CE modulation influences the TPEE flexibility. Even though all samples represent typical stress−strain curves shown by elastomeric materials, modulus, strength and elongation values vary according to compositional modification. For the samples with HS/SS  =  5/5, both elongation and modulus values tend to decrease by the incorporation of SOFA, and the ultimate strength also tends to decrease as the amount of SOFA incorporated increases for both HS/SS  =  5/5 and 6/4 samples. It is interesting to note that the 5/5-SOFA-1 sample bearing 1.3 mol % of SOFA with respect to BDO shows the best ultimate strength (26.97 ± 2 MPa), which are better than that of the 5/5BD sample (24.31 ± 2 MPa) bearing no SOFA, demonstrating that the incorporation of a small amount of more flexible and bulkier CE is helpful in increasing the strength. The broad spectrum of mechanical properties by the incorporation of SOFA occurs due to the variation of the crystallinities of the SOFA-containing TPEEs. 

Shore A and Shore D hardness of TPEEs was measured according to ISO 868. Usually, elastomer hardness is expressed on the Shore A scale, since A is used for flexible type and D for rigid type. Shore A hardness ranges from 96 to 89 for HS/SS = 5/5 samples and 88 to 84 for HS/SS  =  5/5 samples. As the amount of SOFA and SS blocks incorporated increases, it decreases monotonously. Considering Shore A hardness of EPDM for seals and weather-stripping ranges from 50 to 90, conventional TPEEs of their HS higher than 50% are hardly applicable for similar applications. According to our pre-screening results, elastomeric behavior disappeared by simply decreasing HS to less than 40%, demonstrating that sophisticated microstructure modulations are needed to make TPEE more flexible without losing its elastomeric properties, affording a wide range of application to automobile seals and weather-strip.

## 4. Conclusions

A series of TPEEs consisting of PTMEG as a SS block and poly(tetramethylene 2,6-naphthalene dicarboxylate) as an HS block with HS/SS = 50/50 and 40/60 were successfully synthesized, targeting automobile weather-stripping and seal applications. The crystallinity and phase separation behavior could be tuned simply by changing CE compositions to some degree. Thus, the thermal and mechanical properties of TPEEs could be modified in a wide range by sustaining their elastomeric feature intact. In particular, the Shore A hardness ranged from 96 to 84, which is an upper flexible range of TPV.

## Figures and Tables

**Table 1:** Compositions and thermal properties of TPEEs with different hard and soft segments synthesized by using 1,4-butanediol (BDO) or BDO combine with soybean oil-originated fatty acid diethanolamide (SOFA) as chain extender formulation.

HS/SS	Sample Code	*X_SOFA_*^1^ (%) in Feed	*X_SOFA_*^1^ (%) in Polymer	*T*_g1_^2^ (°C)	*T*_g2_^2^ (°C)	*T_m_*^3^ (°C)	*ΔH_m_*^3^ (J/g)	*η*^4^ (dL/g)	*M_v_*^4^ ×10^−4^
50/50	5/5-BDO	0	0	−42	44	195	12.38	1.54	10.1
	5/5-SOFA-1	1	1.3	−47	43	195	10.21	1.65	11.2
	5/5-SOFA-3	3	3.8	−46	43	194	11.85	1.87	13.5
	5/5-SOFA-5	5	5.0	−47	44	188	11.20	2.01	15.0
	5/5-SOFA-10	10	9.2	−48	46	185	10.38	2.06	15.6
	5/5-SOFA-15	15	11.7	−49	42	180	9.26	1.97	14.5
	5/5-SOFA-20	20	14.5	−49	40	176	9.19	1.93	14.1
40/60	4/6-BDO	0	0	−51	41	166	10.98	1.89	13.7
	4/6-SOFA-3	3	4.8	−50	39	164	10.23	1.69	11.7
	4/6-SOFA-5	5	6.1	−49	38	158	9.75	1.74	12.1
	4/6-SOFA-10	10	8.3	−49	39	155	9.62	1.65	11.2

^1^ Mole percentage of SOFA with respect to BDO in feed and in polymer (determined by ^1^H NMR spectroscopy). Note that [CE]/[NDC] ratio is fixed at 1:1.5. ^2^ Glass transition temperatures of soft phase (*T_g1_*) and amorphous part in crystalline hard phase (*T_g2_*). Note that *T_g1_* is almost unchanged according to compositional change. ^3^ Melting (*T_m_*) temperatures, and enthalpies of melting (*ΔH_m_*), measured by DSC. ^4^ Intrinsic viscosity (*η*) and viscosity-average molecular weight (*M_v_*) measured by Ubbelohde-type viscometer.

**Table 2:** The Flory–Huggins interaction parameters between various beads ^1^.

	SS1	HS1	HS2
SS1	0	0.133	0.044
HS1	0.183	0	0.183
HS2	0.044	0.133	0

^1^ SS1, HS1 and HS2 represent beads of SS consisting of structural unit of PTMEG, HSs consisting of structural units formed by the reaction of NDC and BDO, and by NDC and SOFA, respectively. The Flory–Huggins interaction parameter (*χ_ij_*) was obtained by the expression [29]: *χ_ij_* = [*v*(δ_i_ − δ_j_)^2^]/R*T*, where δ_i_ and δ_j_ are solubility parameters of components i and j, respectively, and *v* is the molar volume of beads.

**Table 3:** Mechanical properties of TPEEs with different hard and soft segments synthesized by using BDO or BDO combine with SOFA as chain extender formulation.

HS/SS	Sample Code	Ultimate strength (MPa)	Elongation (%)	Hardness	
				Shore A	Shore D
50/50	5/5-BDO	24.31	1490	96	45
	5/5-SOFA-1	26.97	1318	96	45
	5/5-SOFA-3	23.26	1240	95	43
	5/5-SOFA-5	19.23	1312	94	42
	5/5-SOFA-10	14.09	955	93	41
	5/5-SOFA-15	10.71	754	91	39
	5/5-SOFA-20	7.62	412	89	36
40/60	4/6-BDO	22.01	1543	88	35
	4/6-SOFA-3	19.97	1572	86	32
	4/6-SOFA-5	18.22	1577	85	30
	4/6-SOFA-10	11.93	1370	84	28
